# Effects of herb-partitioned moxibustion for primary dysmenorrhea: A protocol for systematic review and meta-analysis

**DOI:** 10.1097/MD.0000000000021253

**Published:** 2020-07-17

**Authors:** Fengjun Ma, Xiao Yan, Yanpu Yu, Dongqing Du, Shujing Li, Chen Chen, Xiaobin Zhang, Zhibin Dong, Yuxia Ma, Yuning Ma

**Affiliations:** School of Acupuncture-Tuina, Shandong University of Traditional Chinese Medicine, Jinan, Shandong, China.

**Keywords:** herb-partitioned moxibustion, meta-analysis, primary dysmenorrhea, protocol, systematic review

## Abstract

**Background:**

Primary dysmenorrhea (PD) is a cyclic cramp in pelvic which affects the quality of life. Herb-partitioned moxibustion (HPM), a critical component of moxibustion therapy in traditional Chinese medicine, has been used to treat PD. However, there is still a lack of high-quality evidence to support the effectiveness and safety of HPM on patients with PD. The object of this work is to evaluate the efficacy and safety of HPM in the management of PD.

**Methods:**

The Embase, MEDLINE, PubMed, Cochrane Library Central Register of Controlled Trials, China national knowledge infrastructure database, Wan fang database, Chongqing VIP information, and SinoMed will be searched from their inception to Jun 2020. All randomized controlled trials of HPM for the treatment of PD will be included. We will operate article retrieval, duplication removing, screening, quality evaluation, and data analyses by RevMan 5.3 (The Cochrane Collaboration, Oxford, England).

**Results:**

This study will provide a high-quality comprehensive evaluation of the efficacy and safety of HPM for the treatment of PD.

**Conclusion:**

The conclusion of our systematic review will give more convincing evidence to assist clinicians during the decision-making process when dealing with PD.

**Trial registration number:**

10.17605/OSF.IO/UFKNP

## 1 Introduction

Primary dysmenorrhea (PD) is defined as cramping pain in the lower abdomen that occur before or during menstruation in women of childbearing age. It is a common gynecologic disorder that affects between 45% and 95% of menstruating women.^[1]^ The pain of PD usually lasts for 8 to 72 hours and is usually accompanied by other symptoms, such as sweating, headache, vomiting, diarrhea, and tremulousness.^[2]^ Studies have demonstrated that severe menstrual pain is associated with absenteeism from work and limitation of daily activities.^[3]^ It is currently believed that the imbalanced expression of prostanoids and eicosanoids released from the endometrium is the main cause of PD.^[4]^ Thus, nonsteroidal anti-inflammatory drugs (NSAIDs) or oral contraceptives are commonly used in the treatment of PD.^[5]^ However, some patients who use these drugs may suffer from side effects, such as indigestion, nausea, or stomach pain.^[6]^ It is of significance to seek new therapies for the treatment of PD.

Herb-partitioned moxibustion (HPM) is a critical component of moxibustion therapy in traditional Chinese medicine. It treats diseases by placing a piece of herb formula on the patient's acupuncture points and then igniting a moxibustion tube on the herb formula. As a vital part of complementary and alternative medicine, HPM has been used to treat various diseases, including irritable bowel syndrome,^[7]^ retention of urine,^[8]^ and diabetic gastroparesis.^[9]^ Simultaneously, it was also widely used in the treatment of PD. Clinical studies have demonstrated that HPM can reduce menstrual pain and improve quality of life in patients with PD,^[10]^ and its mechanism may be related to the upregulating of 20α-dihydroprogesterone, pregnenolone, prostaglandin E2, and γ-aminobutyric acid.^[11]^ With the publication of clinical trials on HPM for PD, a few studies have shown that HPM has a good clinical effect. However, there is still a lack of high-quality evidence to support the effectiveness and safety of HPM on patients with PD. In this work, we will perform a systematic review to evaluate the efficacy and safety of HPM in the treatment of PD to provide evidence-based guidance for clinical application.

## 2 Material and methods

We will conduct this work following the Preferred Reporting Items for Systematic Reviews and Meta-analysis (PRISMA) Statement.^[12]^ This work has been registered this work at Open Science Framework (OSF, https://osf.io/). The registration DOI of this study is 10.17605/OSF.IO/UFKNP.

### 2.1 Selection criteria

#### 2.1.1 Type of studies

Randomized controlled trials (RCTs) that explore the efficacy and safety of HPM in the treatment of PD will be included. Non-randomized control studies and observational study will be excluded. Studies of animal experiments, review, and case report will be excluded.

#### 2.1.2 Types of patients

Trials involving participants with PD will be included without restrictions of age, economic status, severity of the disease, or education.

#### 2.1.3 Types of interventions and comparisons

Patients in the treatment group must have been treated with HPM therapy or combined with routine treatment recommended by guidelines. Any researches including other Chinese patent medicine, Chinese herb, and acupuncture will be excluded. Studies in which HPM is not used as a major intervention will be excluded.

#### 2.1.4 Types of outcomes

Any outcome that is related to the condition can be included. The primary outcome is pain intensity and the total treatment response rate. The pain intensity is evaluated by the visual analog scale (VAS) or the numerical rating scale (NRS). The secondary outcomes mainly include the overall related symptoms, quality of life, and adverse events.

### 2.2 Search strategy

Two researchers will independently and electronically retrieve the following databases: Embase, MEDLINE, PubMed, Cochrane Library Central Register of Controlled Trials, China national knowledge infrastructure database, Wan fang database, Chongqing VIP information, and SinoMed from their inception to Jun 2020. The Google scholar, Baidu Scholar will be also searched. A search strategy that combines MeSH terms and free words will be adopted. The search strategy in Pubmed is as follows:

- 1#: Search ((((moxibustion[MeSH Terms])) OR (herb partitioned moxibustion[Title/Abstract])) OR (herb partitioned[Title/Abstract]).
- 2#: Search: (((dysmenorrhea[MeSH Terms]) OR (primary dysmenorrhea[Title/Abstract])) OR (algomenorrhea[Title/Abstract])) OR (menalgia[Title/Abstract]).
- 3#: Search: (((((((((clinical trials, randomized[MeSH Terms]) OR (randomized controlled trial[MeSH Terms])) OR (controlled clinical trials, randomized[MeSH Terms])) OR (random allocation[MeSH Terms])) OR (allocation, random[MeSH Terms])) OR (controlled clinical trials, randomized[MeSH Terms])) OR (RCT[Title/Abstract])) OR (controlled clinical trial[Title/Abstract])) OR (randomized[Title/Abstract])) OR (trial[Title/Abstract]).
- #1 and #2 and #3

### 2.3 Study selection and data extraction

#### 2.3.1 Selection of studies

**Figure 1:**
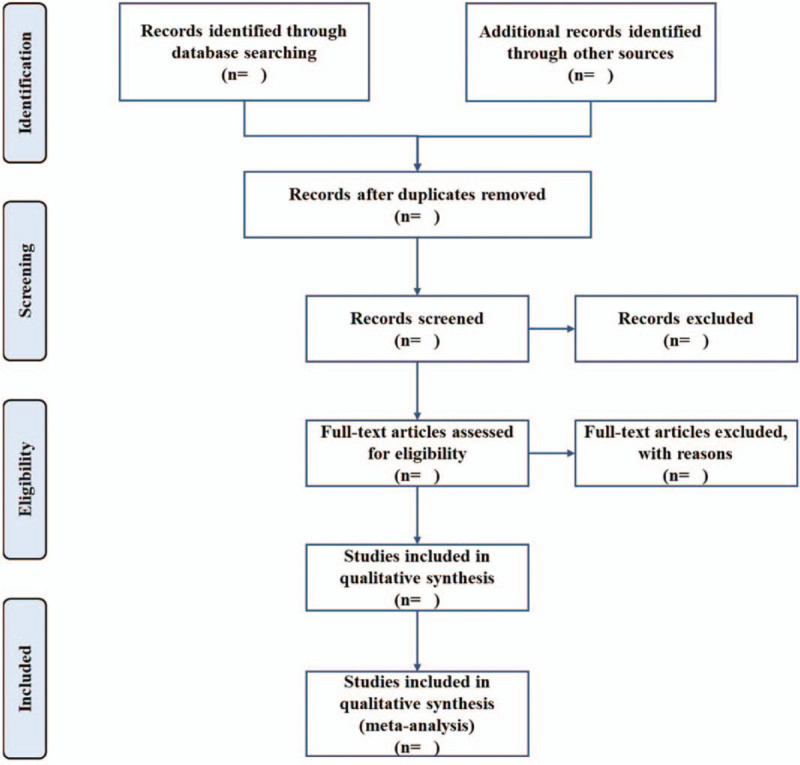
Flow chart of study selection.

Two reviewers will independently review the titles and abstracts following the research criteria and search strategies. The full text will be downed if the studies cannot be identified. Excluded studies will be recorded with the reasons for their exclusion. Any disagreements will be resolved through discussion with other reviewers. The details of the selection process are shown in Figure 1.

#### 2.3.2 Data extraction

Two independent reviewers will carry out data extraction. We will extract the data including the year of study publication, participant characteristics, the severity of disease, sample size, age, dropouts, study period, intervention details, outcomes, and adverse events. When data are missing or unclear, it will be resolved through discussion within the group and contacting the corresponding author.

#### 2.3.3 Assessment of risk of bias

A tool introduced in the Cochrane Handbook for systematic reviews of interventions Version 6 will be used to assess a broad category of biases. We will determine the bias including the following items: random sequence generation, allocation concealment, blinding of the participants and personnel, blinding of the outcome assessments, incomplete outcome data, selective reporting, and other sources of bias. These studies will be evaluated as “Low risk’,” “High risk,” or “Unclear risk.” Inconsistencies will be resolved by discussion within the group.

#### 2.3.4 Measures of treatment effect

For dichotomous data, we will calculate the date and present it by the relative risks with 95% confidence intervals (CIs). For continuous data, we will calculate the effect size using the mean differences with 95% CIs.

#### 2.3.5 Dealing with missing data

The reviewers will attempt to obtain missing data by contacting the corresponding author. If it fails, we will analyze it based on available data.

#### 2.3.6 Assessment of heterogeneity

The heterogeneity between the results included in the study will be calculated by Cochrane X^2^ and I^2^ tests.^[13]^ If *P*≥.05 and I^2^≤50%, the statistical heterogeneity between these studies can be ignored. If *P* < .05 and I^2^ > 50%, it is considered that there is great heterogeneity between these studies.

#### 2.3.7 Assessment of reporting bias

When there are more than 10 included studies, the symmetry of the funnel plot will be drawn to detect reporting biases. The Egger test will be carried out with Stata 12.0 software (Stata Corporation, LLC College Station, TX) for quantitative analysis.^[14]^

#### 2.3.8 Data synthesis

A meta-analysis will be carried out using RevMan 5.3 software (The Cochrane Collaboration, Oxford, England). If there is no statistical heterogeneity among the results, the fixed-effect model will be applied for meta-analysis. If there is statistical heterogeneity between the results, a random-effect model will be used. If there is obvious clinical heterogeneity, subgroup analysis or sensitivity analysis will be conducted.

#### 2.3.9 Subgroup analysis

Subgroup analysis will be conducted based on different interventions, controls, durations of treatment, and outcome measures.

#### 2.3.10 Sensitivity analysis

We will conduct a sensitivity analysis to confirm the robustness of our findings. The principal decision nodes conclude methodological quality, sample size, and the effect of missing data. The meta-analysis will be repeated and studies of lower quality will be excluded. Therefore, we will be able to assess the impact of low-quality studies on the overall results.

#### 2.3.11 Evaluation of the evidence quality

To assess the quality of evidence, the Grading of Recommendations Assessment, Development, and Evaluation will be used. In the Grading of Recommendations Assessment, Development, and Evaluation system, the quality of evidence can be defined as high, moderate, low, and very low.

#### 2.3.12 Ethics and dissemination

This systematic review will not require ethical approval because there are no data used in our study that are linked to individual patient data. The results will be disseminated only in a peer-reviewed publication.

## 3 Discussion

PD is a cyclic cramp in pelvic which affects the daily activity and quality of life. Therefore, effective intervention should be conducted in the management of PD. As a characteristic of external therapy in Chinese medicine, HPM was used to treat PD through regulating the reproductive endocrinal level.^[15]^ Nevertheless, currently, no systematic review related to HPM for PD has been published. Therefore, we will conduct this systematic review to verify the effectiveness of HPM in the treatment of PD. We hope that this review will give more convincing evidence to assist clinicians during the decision-making process when dealing with PD.

## Author contributions

**Data curation:** Xiao Yan

**Formal analysis:** Yanpu Yu and Xiaobin Zhang

**Methodology:** Dongqing Du and Zhibin Dong

**Project administration:** Yuxia Ma and Yuning Ma

**Resources:** Fengjun Ma and Xiao Yan

**Software:** Fengjun Ma, Xiao Yan, and Chen chen

**Visualization:** Yanpu Yu, Shujing Li

**Writing – original draft:** Fengjun Ma and Yuxia Ma

**Writing – review & editing:** Yuning Ma
